# Characterization of Global Research Trends and Prospects on Single-Cell Sequencing Technology: Bibliometric Analysis

**DOI:** 10.2196/25789

**Published:** 2021-08-10

**Authors:** Quan Wang, Ke-Lu Yang, Zhen Zhang, Zhu Wang, Chen Li, Lun Li, Jin-Hui Tian, Ying-Jiang Ye, Shan Wang, Ke-Wei Jiang

**Affiliations:** 1 Department of Gastroenterological Surgery Peking University People’s Hospital Beijing China; 2 Laboratory of Surgical Oncology Beijing Key Laboratory of Colorectal Cancer Diagnosis and Treatment Research Peking University People’s Hospital Beijing China; 3 Evidence-Based Nursing Center School of Nursing Lanzhou University Lanzhou China; 4 Department of Gastrointestinal Surgery Shandong Provincial Hospital Affiliated to Shandong First Medical University Jinan China; 5 Department of Breast Surgery Shanghai Cancer Center Fudan University Shanghai China; 6 Evidence-Based Medicine Center School of Basic Medical Sciences Lanzhou University Lanzhou China

**Keywords:** single-cell sequencing, bibliometric analysis, cancer, cancer genomics, bioinformatics, cancer subtyping, tumor dissociation, tumor microenvironment, precision medicine, immunology, development trends, hotspots, research topics, Web of Science, CiteSpace, VOSviewer, network

## Abstract

**Background:**

As single-cell sequencing technology has been gradually introduced, it is essential to characterize global collaboration networks and map development trends over the past 20 years.

**Objective:**

The aim of this paper was to illustrate collaboration in the field of single-cell sequencing methods and explore key topics and future directions.

**Methods:**

Bibliometric analyses were conducted with CiteSpace and VOSviewer software on publications prior to November 2019 from the Web of Science Core Collection about single-cell sequencing methods.

**Results:**

Ultimately, we identified 2489 records, which were published in 495 journals by 14,202 authors from 1970 institutes in 61 countries. There was a noticeable increase in publications in 2014. The United States and high-income countries in Europe contributed to most of the records included. Harvard University, Stanford University, Karolinska Institutes, Peking University, and the University of Washington were the biggest nodes in every cluster of the collaboration network, and SA Teichmann, JC Marioni, A Regev, and FC Tang were the top-producing authors. Keywords co-occurrence analysis suggested applications in immunology as a developing research trend.

**Conclusions:**

We concluded that the global collaboration network was unformed and that high-income countries contributed more to the rapidly growth of publications of single-cell sequencing technology. Furthermore, the application in immunology might be the next research hotspot and developmental direction.

## Introduction

A single cell is regarded as the fundamental unit of an organism. Moreover, there are approximately 10^14^ single cells that comprise the complex tissues and organs of *Homo sapiens* [1]. In order to study genomic or transcriptome information, most published studies to date have collected data by analyzing bulk tissue samples and true genomic information representing the average of millions of cells [2]. Accordingly, because of cell-to-cell variability, traditional sequencing methods only provide the average diversity of cells, instead of obtaining information on entire cellular heterogeneity [3]. When facing the complexity of disease, it is difficult for these averaged data sets to represent cell-to-cell variations; therefore, it is difficult for researchers to identify rare cells, including cancer stem cells, that play a key role in cancer progression. To avoid the weakness and limitations of bulk sequencing technologies, the single-cell method offers a novel possibility that focuses on the single-cell level [4], with detailed and comprehensive studies of individual cells rather than traditional analysis of bulk tissue. The first step of sequencing a single cell, however, involves capturing individual cells; therefore, it is of utmost importance to establish various methods for isolating single cells from abundant populations or rare single cells (<1%) from typical populations. Several approaches have been well-established, including mouth pipetting, serial dilution, robotic micromanipulation, flow-assisted cell sorting, microfluidic platforms, and laser-capture microdissection, however, the isolation of rare single cells (<1%) is far more challenging [2].

With increasing developments of high-throughput sequencing technologies over the past couple of decades, an increasing number of commercial platforms have been introduced for experimental and clinical applications, and thousands of genomes from numerous species have been sequenced [3]. Researchers now have a wealth of data to explore genomic or transcriptome information. Single-cell sequencing refers to the sequencing of a single-cell genome or transcriptome, to reveal cell population differences and cellular evolutionary changes [5]. Furthermore, these commercial methods interrogate the single-cell genome, transcriptome, and epigenome. For example, since the introduction of the first single-cell RNA-sequencing technique in 2009 [6], single-cell RNA sequencing has become a powerful and useful approach to study individual cell transcriptomes on a large scale [7], similar to the technology of multiomics sequencing [8]. When it comes to the applications of single-cell sequencing techniques, the important role of single-cell sequencing in various fields, including oncology [9], microbiology [10], neurology [11,12], reproduction [13], and immunology [14] has been highlighted. These rapidly developing methods have and will continue to lead to new discoveries in many scientific fields.

Recently, because single-cell sequencing technology has been gradually introduced for clinical applications, there has been an increase in research papers published online on this topic. There are more than 70 related records in this field, published online each month on PubMed, and these are of a variety of publication types. Some papers offered a brief introduction of single-cell sequencing technology [15], whereas others focused on investigating its applications [16]. In some reviews [7,17], the current strengths and disadvantages of single-cell sequencing technology were summarized. Published original articles updated or simplified the algorithms of computational methods [18].

Based on this, it is important for us to better understand and learn more from the developmental trends and novel advancements of single-cell sequencing techniques macroscopically; however, rapidly learning about the current landscape of specific biomedical research is still a challenge. Fortunately, the emergence of bibliometric analysis provides an approach to statistically and quantitatively visualize evidence according to the information in published records in a specific field [19,20], which includes data, such as keywords, citation reports, authors, affiliated countries, institutions, and journals, from a large number of published studies. To the best of our knowledge, no specific study has focused on the characterization of research hotspots, global research collaborations, and developmental trends of single-cell sequencing techniques. To characterize current evidence and establish future research directions, it is essential to perform bibliometric analysis to map global collaboration patterns and developmental trends of published single-cell sequencing methods.

The aims of this bibliometric analysis were to map this research landscape through analysis of the information in published records.

## Methods

### Data Source and Search Strategy

We searched the Web of Science Core Collection (WoSCC) without publication date restrictions on November 12, 2019. We updated the database search on January 29, 2020, to complete data retrieval for the year of 2019; however, only the number of records published annually was updated.

The search was performed using the following keywords and terms: (“single-cell RNA sequencing” OR “scRNA-seq” OR “single-cell RNA-seq” OR “single-cell sequencing” OR “single-cell transcriptomic” OR “single-cell ATAC” OR “single-cell RNA-sequencing” OR “single-cell omics sequencing” OR “scRNA seq”). The search strategy was peer-reviewed and guided by TJH (who has over 10 years of experience as an information specialist). In this study, only publications in English were included; however, there was no restriction on data category.

### Inclusion and Exclusion Criteria

Inclusion criteria were published records, including articles, comments, reviews, letters, and brief introductions on single-cell sequencing techniques. We included the records that, not only focused on the single-cell sequencing technology after the paper of single-cell RNA-sequencing published in 2009 [6], but also, generally used the single-cell sorting methods such as Flow sorting in early time. We excluded duplicates, conference abstracts, and manuscripts in a language other than English, for consistent and accurate information collection from multianalysis results based on published records. Two groups of reviewers (WQ/WZ/JKW and YKL/LL) independently screened the titles and abstracts to select the articles after standard selection training. Full-texts were retrieved when necessary. Disagreements were discussed and solved between the 2 groups.

### Social Network Map Software

CiteSpace (version 5.3.R4) was used for social network analysis of developmental dynamics, future trends, hotspots, and key points in scientific literature of a specific topic [21]. Burst detection, to identify keywords and references that appear with an abrupt change in frequency at a certain period, were considered to be hot keywords or references at that point in time. Clustering (or co-citation, ie, both A and B are cited by C) analysis of references was also conducted [21]. We used VOSviewer (version 1.6.9; Leiden University) to visualize the collaborations between authors of a list of publications as well as those between countries, institutions, and high frequency keywords [22]. One classification method uses a metric based on the number of co-authored articles, which allowed authors, institutions, countries or keywords to be clustered, where those belonging to the same group cooperated more with each other [23]. In network maps, unequal-size nodes with different colors represent differences in the number or frequency of published records in clusters among the same research topics [21]. Lines between nodes indicate the strength of collaborations (the stronger the collaboration, the thicker the line) [24]. We also used overlay visualization, in which the color of the node represents the average year in which each author, institution, country or keyword was used [25].

Data were saved as *Plain Text* with *Full Record and Cited References* in WoSCC and imported into CiteSpace.

### Statistical Analysis

SPSS software (version 22.0; IBM Corp) and Origin (version 2018; OriginLab Corp) were used for data analysis. Continuous variables were presented as mean and standard deviation or median and interquartile range, and categorical variables were expressed using frequencies and percentages.

## Results

### Search Results

**Figure 1:**
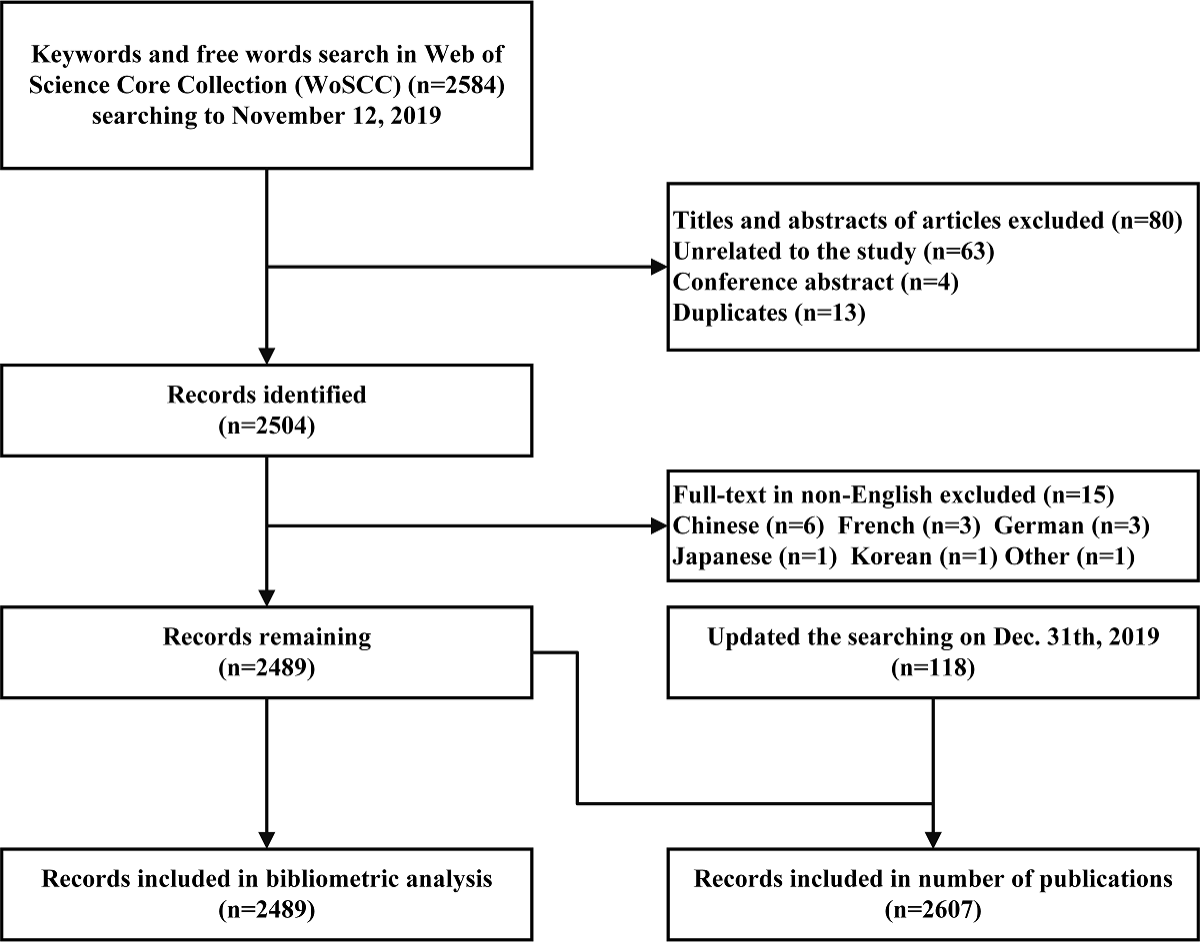
Record identification and selection.

A total of 2584 publications were identified from WoSCC. After screening titles and abstracts, duplicates, unrelated topics, and conference abstracts (n=80) or non-English publications (n=15) were excluded. Finally, 2489 studies were included for bibliometric analysis (Figure 1); 2607 was identified as the number of records published.

### Global Publication Trends

Papers published over the past of two decades (Table 1 and Table 2) were primarily classified into 7 types. Most papers were original articles (1858/2607, 71.27%), and there were 388/2607 meeting abstracts (14.84%) and 217/2607 reviews (8.32%). Most publication types were articles, meeting abstracts, and reviews, which can greatly reflect the development trends and changes in single-cell sequencing technology. The first publication on single-cell sequencing technology was in 2001 [26], which reported single-cell sequencing of dinoflagellate (Class Dinophyceae) nuclear ribosomal genes. In addition, there were only was only 1 to 3 publication per year before 2010. From 2010 to 2019, the number of publications showed a noticeable upward trend, with increases since the year 2014 (n=59), reaching 987 publications in 2019.

**Table 1:** Number of published records per year.

Year	Records, n
2001	1
2002	0
2003	2
2004	0
2005	1
2006	1
2007	1
2008	2
2009	2
2010	3
2011	10
2012	21
2013	35
2014	59
2015	127
2016	225
2017	406
2018	722
2019	989
Total	2607

**Table 2:** Percentage of published records by publication type.

Publication type	Records (n=2607), n (%)
Article	1858 (71.27)
Meeting abstract	388 (14.84)
Review	217 (8.32)
Editorial material	97 (3.72)
Correction	29 (1.11)
Letter	13 (0.54)
News item	5 (0.19)

### Countries and Districts

In total, 61 countries contributed to publications on single-cell sequencing technology worldwide. The results demonstrated that 7 countries published more than 100 records (Table 3), and the United States (1454/2489, 58.4%), with more than half of all identified records, published the most. The top 2 to 7 were China (414/2489, 16.6%), the United Kingdom (305/2489, 12.3%), Germany (225/2489, 9.0%), Sweden (134/2489, 5.4%), Japan (108/2489, 4.3%), and Switzerland (104/2489, 4.2%). Although the number of documents published by Australia, Canada, and the Netherlands was less than 100, no significant differences were observed (*P*=.10), and the contribution was almost equal to that of Japan and Switzerland. In addition, the cooperation among studies in China was a little less than that in the United Kingdom. However, Sweden had the highest average number of citations (52.0 times), followed by Canada (37.4 times), the United Kingdom (29.6 times), the United States (27.7 times), Australia (24.7 times), and Germany (23.9 times).

**Table 3:** Distribution by country.

Rank	Country	Documents, n (%)	Citations, n	Average citations	Total link strength
1	USA	1454 (58.4)	40337	27.7	549
2	China	414 (16.6)	6537	15.8	206
3	UK	305 (12.3)	9026	29.6	214
4	Germany	225 (9.0 )	5384	23.9	162
5	Sweden	134 (5.4)	6973	52.0	88
6	Japan	108 (4.3)	1454	13.5	68
7	Switzerland	104 (4.2)	1802	17.3	72
8	Australia	99 (4.0)	2442	24.7	70
9	Canada	85 (3.4)	3177	37.4	53
10	Netherlands	85 (3.4)	1330	15.7	67

**Figure 2:**
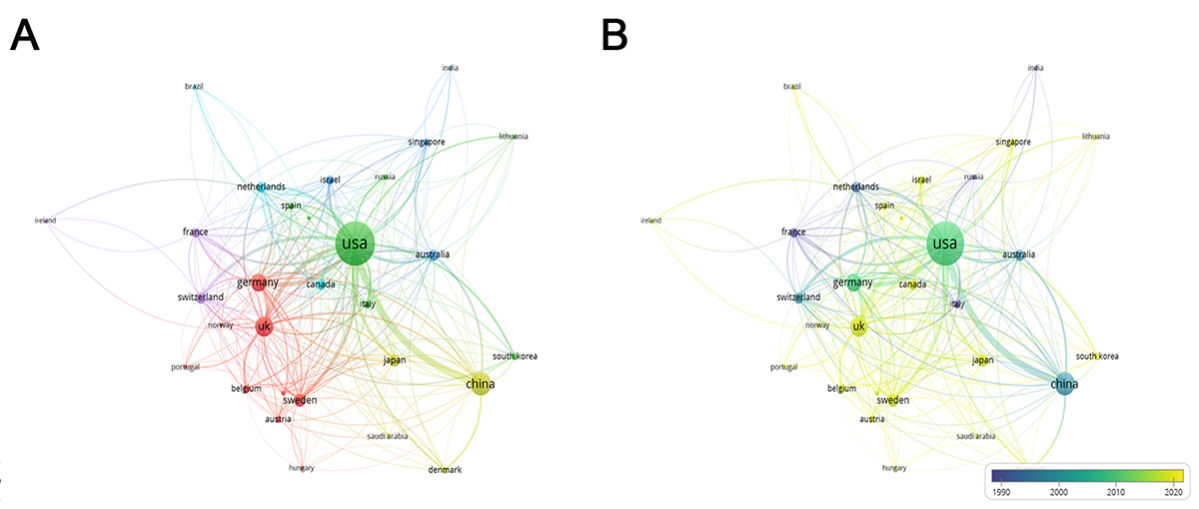
Distribution of countries and regions. (A) The network map of countries (TOP30). (B) Dynamics and trends of countries/regions over years (TOP30).

For the top 30 most prolific countries, which formed 6 clusters, a network map was created. There were active collaborations among these countries, especially between the United States and China (Figure 2A). From the dynamics and trends, it can be seen that the United States, China, Germany, Switzerland, France, Australia, and the Netherlands have carried out studies on single-cell sequencing technology dating back to 2009. After 2015, many other investigators worldwide started to pay more attention to this field (Figure 2B).

### Universities and Institutions

**Figure 3:**
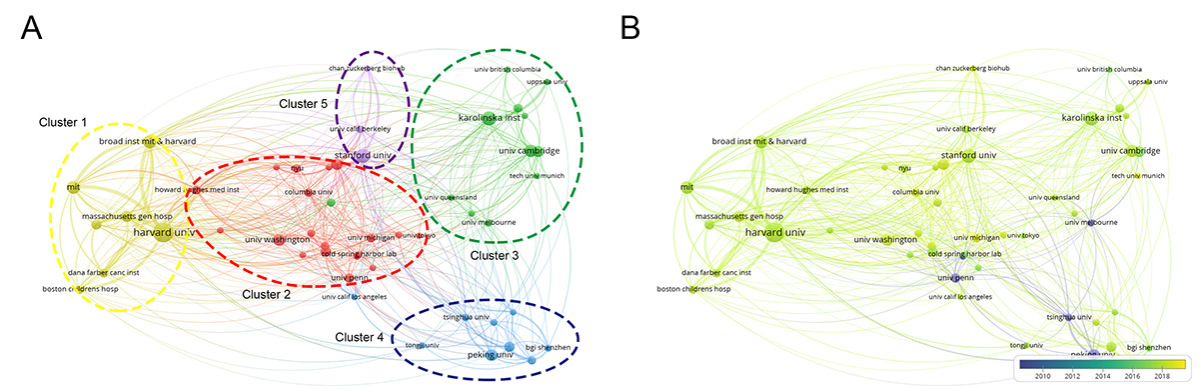
Distribution of universities and institutions. (A) The network map of universities and institutions (TOP50). (B) Dynamics and trends of universities and institutions over time (TOP50).

A total of 1970 universities or institutions made contributions to single-cell sequencing technology; extensive cooperation network analysis was carried out among universities or institutions. In a network map and overlay visualization of institutions with the top 50 frequency, 5 clusters were formed (Figure 3A), and Harvard University (n=226, 9.1%, Cluster 1), Stanford University (n=114, 4.6%, Cluster 5), Karolinska Institute (n=111, 4.5%, Cluster 3), Peking University (n=81, 3.3%, Cluster 4), and the University of Washington (n=77, 3.1%, Cluster 2) were the biggest nodes in every cluster, respectively. Cluster 1 was the biggest cluster, which contained 19 nodes that represented its related different universities or institutions, while Cluster 5 was the smallest, which included 3 nodes. The other clusters included 7 nodes (Cluster 2), 12 nodes (Cluster 3), and 9 nodes (Cluster 4). Furthermore, the results also showed that 4 universities contributed to primary and basic research on these topics earlier in time and started earlier on this field before 2010, which included Peking University, University of Melbourne, University of Penn, and the Tsinghua University. Since then, these studies have gradually gained popularity among other universities and institutes (Figure 3B).

### Authors

**Figure 4:**
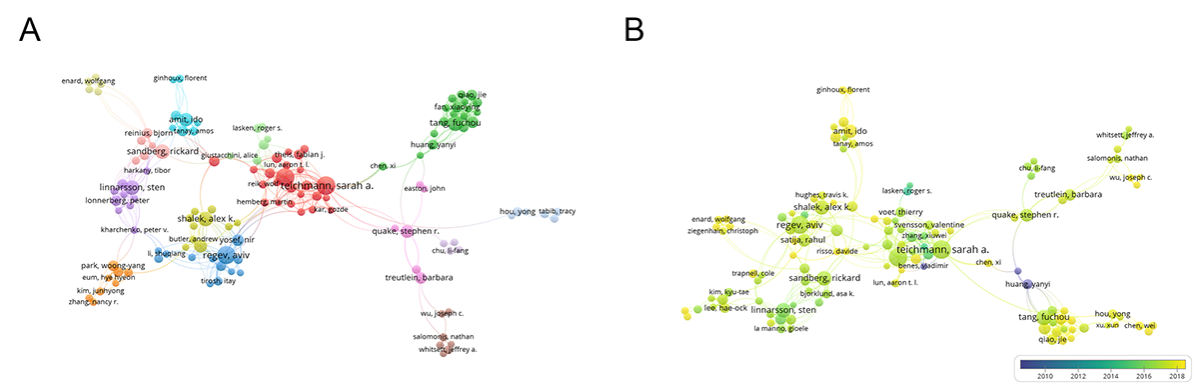
Distribution of authors. (A) The network map of authors (TOP200). (B) Dynamics and trends of authors over time (TOP200).

A total of 14,202 authors contributed to single-cell sequencing technology publications. The network map and overlay visualization of the top 200 cooperatively productive authors formed several clusters (Figure 4). Of these authors, 5 scientific teams contributed to most publications worldwide. The biggest 3 nodes were SA Teichmann (40/14,202, 1.6%) and JC Marioni 38/14,202, 1.5%), who had the biggest cluster and were from the University of Cambridge. In addition, A Regev (38/14,202, 1.5%) had the second biggest cluster and was from Massachusetts Institute of Technology. The fourth node, FC Tang, represented a group from Peking University (26/14,202, 1.0%), and 2 authors, R Sandberg and S Linnarsson from Sweden contributed to 49 records in total. Moreover, I Amit, from Israel, published 21 records to date (Multimedia Appendix 1).

### Journals

This study showed that a total of 495 journals published articles about single-cell sequencing technology. The results showed that the most productive journal was *Nature Communications* (127/14,202, 5.1%), the most cited journal was *Nature Methods* (55/14,202, 2.2%, 1764 citation times in total), and the journal with the highest average citation was *Nature Biotechnology* (28/14,202, 1.1%, average citation was 56.5 times). The top productive journals, top cited journals, and journals of top average citations are listed (n=20) in Multimedia Appendix 2.

### Top 10 Citations of Included Records

Of the 2489 papers, the top 10 publications ranked by citation are listed in Table 4. The first paper was published in the *Journal of Computational Biology* by A Bankevich et al in 2012 [27] and reported a new genome algorithm and its applications to single-cell sequencing, with 5668 citations, which was much higher than that of the second paper (1307 citations) [28]. Of the 10 records, 4 were published in *Science*, and the topics included intratumoral heterogeneity in primary glioblastoma [29], metastatic melanoma [30], and cell types in the mouse cortex and hippocampus revealed by single-cell RNA-sequencing [31]. The latest paper [30], which was published in 2016, has been cited 652 times (from 2016 to 2019).

**Table 4:** Distribution by top 10-record co-citations.

Rank	Top-cited record	Number of citations	Title	Journal
1	A Bankevich, 2012 [27]	5668	SPAdes: A New Genome Assembly Algorithm and Its Applications to Single-Cell Sequencing	*Journal of Computational Biology*
2	N Navin, 2011 [28]	1307	Tumour evolution inferred by single-cell sequencing	*Nature*
3	AP Patel, 2014 [29]	1168	Single-cell RNA-seq highlights intratumoral heterogeneity in primary glioblastoma	*Science*
4	Y Peng, 2012	1039	IDBA-UD: a de novo assembler for single-cell and metagenomic sequencing data with highly uneven depth	*Bioinformatics*
5	A Zeisel, 2015 [31]	923	Cell types in the mouse cortex and hippocampus revealed by single-cell RNA-seq	*Science*
6	C Trapnell, 2014	773	The dynamics and regulators of cell fate decisions are revealed by pseudotemporal ordering of single cells	*Nature Biotechnology*
7	S Picelli, 2014	734	Full-length RNA-seq from single cells using Smart-seq2	*Nature Protocols*
8	I Tirosh, 2016 [30]	652	Dissecting the multicellular ecosystem of metastatic melanoma by single-cell RNA-seq	*Science*
9	DA Jaitin, 2014	615	Massively Parallel Single-Cell RNA-Seq for Marker-Free Decomposition of Tissues into Cell Types	*Science*
10	R Satija, 2015	605	Spatial reconstruction of single-cell gene expression data	*Nature Biotechnology*

### Co-citation References

**Figure 5:**
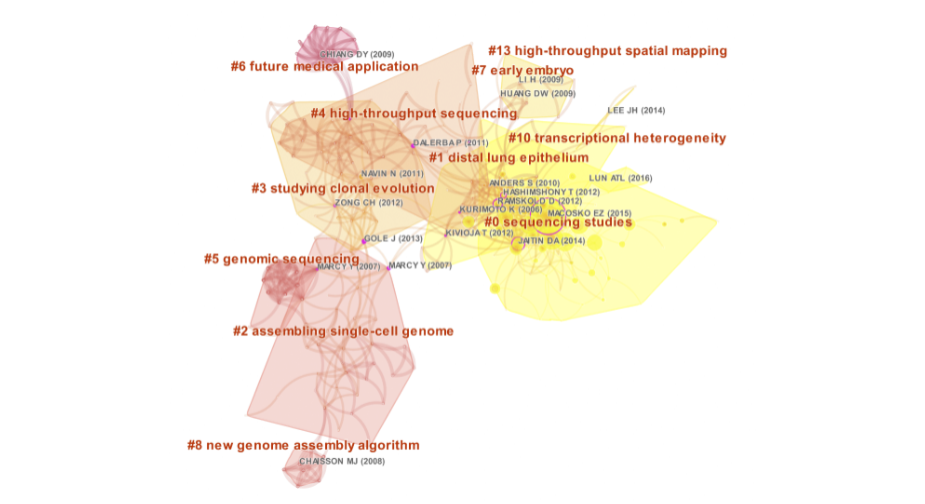
The co-citation network of references.

We used CiteSpace to visualize the co-citation network of references, which were divided into 11 co-citation clusters (Figure 5). The clusters were listed from 2008 to 2016: “transcriptional heterogeneity” (Cluster 10, n=4), “sequencing studies” (Cluster 0, n=53), “high-throughput spatial mapping” (Cluster 13, n=3), “distal lung epithelium” (Cluster 1, n=37), “studying clonal evolution” (Cluster 3, n=33), “early embryo” (Cluster 7, n=9), “assembling single-cell genome” (Cluster 2, n=34), “high-throughput sequencing” (Cluster 4, n=27), “new genome assembly algorithm” (Cluster 8, n=7), “genomic sequencing” (Cluster 5, n=14), and “future medical applications” (Cluster 6, n=13).

The top 36 references with the strongest citation bursts are presented in Multimedia Appendix 3. The first reference with a citation burst appeared in 2011, and most of the bursts appeared between 2011 and 2016. Only 1 reference [32] appears with a burst in the last 3 years.

### Analysis of Keywords

**Figure 6:**
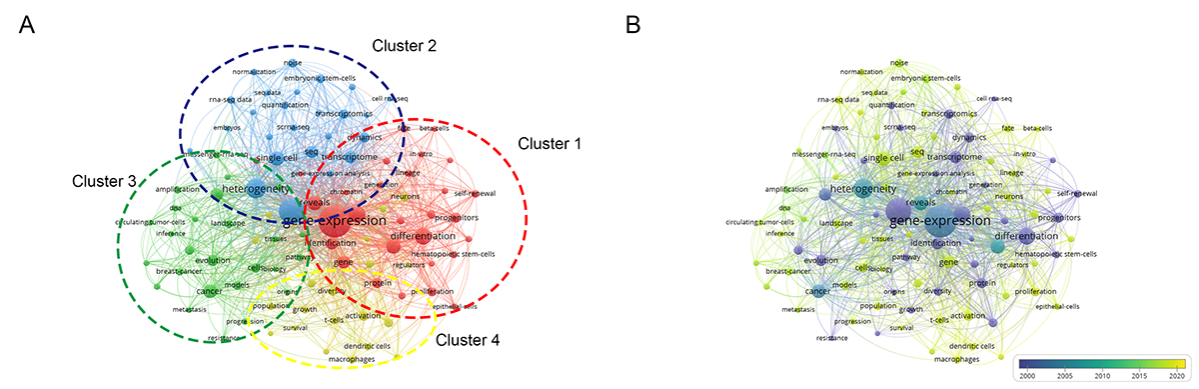
Distribution of 100 frequency keywords. (A) Network map of the frequency keywords(TOP100). (B) Dynamics and trends of the frequency keywords over times(TOP100).

From the 2489 published records, a total of 6012 keywords were extracted. The network map of the top 100 frequency keywords was clustered and formed 4 clusters (Figure 6A), and the biggest 3 nodes were “gene-expression,” “RNA-seq,” and “heterogeneity.” Moreover, the overlay visualization of the top 100 frequency keywords between 2001 and 2020 is shown in Figure 6B.

Cluster 1, with the biggest node “gene-expression,” represented the primary research points of single-cell sequencing, such as stem cells, self-renewal, progenitors, lineage, differentiation, protein and identification, which might be based upon next generation sequencing and single cell isolation methods. Cluster 2 represented improvements in computer algorithms for sequencing data and advancements in single-cell sequencing methods, for instance, keywords such as heterogeneity, transcriptome, RNA-sequencing data, dynamics, noise, and normalization, especially, the stem cells of embryos might be paid more attention to in this cluster. Cluster 3, considered as the currently being developed field in this topic, and could be named the research hotspots of single-cell sequencing. Moreover, the biggest node of Cluster 3 was marked as cancer, which was related to many keywords, including circulating tumor-cells, origin, evolution, landscape, metastasis, resistance, and progression. In addition, it was also indicated that these topics on breast cancer might stay in a leading position and published more papers. Regarding the future direction of single-cell sequencing technology, Cluster 4 was entitled developmental trends of immunology in single-cell sequencing, and keywords included dendritic cells, T-cells, macrophages, and diversity, which might be related to tumor survival, neuronal growth, and activation of regulators. Moreover, the results in Figure 6B showed that the biggest nodes, such as “gene-expression,” “RNA-seq,” and “heterogeneity,” emerged around the year 2005. Subsequently, the frequency of many keywords, including “single-cell,” “noise,” “seq,” and “gene” gradually increased after 2010 and rapidly gained interest from 2015 to 2020.

The data showed that all keywords with citation bursts first appeared in 2001 (Multimedia Appendix 4). Similarly, breast cancer was a primary research hotspot from 2011 to 2017. Furthermore, at the beginning of 2013, many keywords related to computer algorithms, such as “noise” and “evolution,” displayed the strongest citation bursts. Regarding the keyword single-cell sequencing, the time-period of the strongest citation bursts was from 2015 to 2017.

Table 5 summarizes occurrence and the total link strength of the top 30 keywords. Higher keyword occurrences indicated the primary research hotspot was single-cell sequencing applications, including “heterogeneity,” “differentiation,” “genome,” and “transcriptome” of “single cells,” which mainly covers the field of stem cells, such as embryonic stem cells, and the field of cancer. Furthermore, the dynamics, landscape, and diversity of single cells maintained their popularity and showed a stronger total link strength among these 30 keywords.

**Table 5:** Occurrences and total link strength of top 30 keywords.

Rank	Keyword	Occurrences	Total link strength
1	Gene-expression	857	851
2	RNA-seq	563	559
3	Heterogeneity	331	325
4	Stem-cells	235	234
5	Differentiation	231	231
6	Single cell	177	177
7	Reveals	170	170
8	Cancer	155	154
9	Transcriptome	135	135
10	Gene	133	132
11	Mouse	133	132
12	Genome	132	130
13	Identification	125	125
14	Seq	125	122
15	Evolution	115	115
16	Progenitors	93	93
17	Protein	92	92
18	Dynamics	90	90
19	Activation	83	83
20	Transcriptomics	80	78
21	Cells	77	77
22	Receptors	73	73
23	Noise	70	70
24	Mutations	69	69
25	Proliferation	67	67
26	Embryonic Stem-cells	66	66
27	Landscape	65	65
28	Neurons	65	65
29	Models	64	64
30	Diversity	63	63

## Discussion

### Principal Results

In this study, we summarized research collaborations, new development, and research trends of single-cell sequencing worldwide. In particular, bibliometric analysis offers a new possibility of visualizing current hotpots and trends in this field based on the information of published records [19,20]. We were able to analyze the most productive authors and institutions, by journal and by co-citation references, and we illustrated the different research groups of authors with collaboration networks in the past two decades. In total, 2489 records were identified, published in 495 journals by 14,202 authors from 1970 institutes in 61 countries. The United States had the highest absolute productivity ranking, followed by China and Japan (East Asia), the United Kingdom, Germany, other European countries (Sweden, Switzerland, the Netherlands), and Australia and Canada. Taken together, these results indicated that single-cell sequencing technology developed rapidly and spread globally in the past 5 years (after 2015), which might be influenced by the amount of funding available for single-cell sequencing projects in each country. Li et al [33] investigated the distribution and development of single-cell sequencing in China, the United Kingdom, and the United States, focusing on government support, and concluded that there was a gap between high-income and low-income countries with respect to the amount of funding and number of projects. When compared with China, the amount of funding and the number of projects for single-cell sequencing in the United States and the United Kingdom had increased dramatically before 2016 (915 projects, cumulative funding of US $539 million). On the other hand, the later the starting time of foundation support for studies on single-cell sequencing, the less applications of this technology.

After the first record on single-cell sequencing, on the nuclear ribosomal genes of dinoflagellate (Dinophyceae), was published in 2001 [26], the number of publications was sporadic (1 to 3 annually until 2010). With the advancement of high-throughput sequencing and new computer algorithms, the development trend of publications changed from 2010 to 2019, showing a clear upward trend since 2014. However, as the development of single-cell sequencing technology has lasted for almost 20 years, several studies represent basic milestones of single-cell sequencing [2]. For example, the advancement of next-generation sequencing platforms in 2005 [34] offered a better approach for primary single-cell sequencing for isolated single cells. Subsequently, Tang et al [6] were the first to introduce RNA transcriptome sequencing of a mammalian cell in 2009, and Navin et al [28] published the first genomic sequencing of single human cells in 2011. Furthermore, several representative papers were published in the subsequent 3 years, including not only exome single-cell DNA sequencing [35] and its related single-cell DNA sequencing of sperm cells [36], neurons [37], circulating tumor cells [38], and the microbial tree [39], but also, the first single-cell sequencing of the RNA of immune cells [40] and of epigenomes [41]. Certainly, single-cell sequencing methods have supported advances in other technologies, including multiomics sequencing [8]; methods have led to novel discoveries in many scientific fields, such as tumor research [9], microbiology [10], neurology [11,12], reproduction [13], and immunology [14], which could explain the recent exponential increase in the number of publications.

While the United States published the most records worldwide (1454/2489, 58.42%), Sweden had the highest citation average (52.04 times). Citations differed from the number of documents, as did the cooperation of countries and regions: United States, China, United Kingdom, and Germany showed the strongest cooperation, with higher total link strengths. This was mainly because studies on single-cell sequencing technology were published earlier in these countries, which was indicated by the dynamics and trends of countries and regions over the years (Figure 3B). We believe that publications from Sweden had deeper impacts on the single-cell sequencing technology, although the field seems to be small and young enough that most of the findings can be entirely due to individual pioneering researchers. Furthermore, we also found that Karolinska Institutes, from Sweden, was one of the biggest nodes in its cluster of collaboration network (Figure 4A; Cluster 3), which might provide an indirect explanation. Similarly, the analysis of institutions and authors almost matched the distribution by countries and districts. Of the 1970 universities or institutions, the institutes from United States (Harvard University, Stanford University, University of Washington, and University of Penn), European universities (Karolinska Institute and Cambridge University), and Chinese institutes (Peking University and Tsinghua University), as well as the University of Melbourne in Australia, have taken leading places in single-cell sequencing technology, with steady collaborations in global groups. Moreover, of the 14,202 authors who contributed to publications, the top 200 authors were situated in the collaboration networks that account for large proportions of research, mainly from the above-mentioned countries and institutes.

Single-cell sequencing avoids the drawbacks of bulk experiments, which only demonstrate average gene expression, and it can be used to study real differences and evolutionary changes in single cells [7]; therefore, it has gained the popularity and interest of the editors of many journals. Of the 495 journals, 20 journals contributed the most. The top journals were *Nature Communications* (most papers with 127 papers), *Nature Methods* (most cited with 1764 citations), and *Nature Biotechnology* (highest average citations with 56.46 citations per paper). Moreover, of included records, all the top 10 most cited papers were published in the top journals. In addition, the research topics of these top-cited records reported or introduced a new genome algorithm and application of single-cell sequencing [27-31], which together have been cited more than 600 times in the past of 5 years. In co-citation analysis, knowledge on “clonal evolution,” “early embryo,” “assembling single-cell genome,” “high-throughput sequencing,” and “new genome assembly algorithm” were the basis of single-cell sequencing research, and most references with citation bursts appeared between 2011 and 2016.

Of 4 clusters in visualization maps to explore key topics and future directions, Cluster 1 represented the basis of single-cell sequencing (next generation sequencing and single cell isolation methods researched earlier), while Cluster 2 represented the development of computer methods and algorithms. Cluster 3 showed current research hotspots of single-cell sequencing, such as applications in cancer research, with keywords including, circulating tumor-cells, origin, evolution, landscape, metastasis, resistance, and progression, and Cluster 4 showed that immunology will be the next hotspot of single-cell sequencing technology research, with keywords including dendritic cells, t-cells, macrophages, and diversity. In particular, in immunology, neuronal growth and the activation of regulators have been studied in recent years, rapidly gaining interest from 2015 to 2020.

### Limitations

Our study had several limitations. First, we only searched records from the WoSCC and only included records in English, which might result in selection bias. However, the number of included records in our study was large enough to represent the current evidence landscape of single-cell sequencing technology [42]. Second, although the data had been manually standardized, bias might still exist due to the authors with the same name or the keywords of various expressions. Third, by using software, we might have overlooked some information, which may result in errors in data analysis, even if there are some reasonable and unavoidable differences on the same outcomes between CiteSpace and VOSviewer. This was the first study to perform bibliometric analysis on single-cell sequencing technology research, to identify collaboration networks among authors, countries, and institutions and illustrate developmental trends, current hotspots, and future directions in this field.

### Recommendations for Future Work

Single-cell sequencing methods have addressed the drawbacks of averaged gene expression in bulk experiments and provide a possible approach to understanding biological diversity and rare cells. Over the past 10 years, the applications have had a broad impact in many fields, which emerged as delineating cell diversity, tracing cell lineages, classifying cell types, and profiling rare cells [2]. We only selected publications related to immunotherapy in the entire year of 2019, to form and label clusters (Multimedia Appendix 5). Eventually, the formed clusters illustrated that current hotspots in immunology involve at least 5 topics. Cancer immunotherapy is an exciting topic, for which single-cell sequencing methods have great potential for investigating the heterogeneity of intratumor immunity and other topics such as immune cell involvement of breast cancer therapy, role of immune cells in nervous system, formation and differentiation of immune cells, and functions of immune cells in the tumor microenvironment. Cancer therapy research to clarifying the functions of T-cells and macrophages, understand the antigenicity in intratumor heterogeneity, and study the role of clonal diversity in transformation, invasion, and evolution of chemoresistance would open new avenues for preventing cancer in the future. Cancer immunotherapy has revolutionized cancer treatment, and a detailed immune cell atlas at single-cell resolution could facilitate a comprehensive understanding the immunity landscape in tumor microenvironments [43] Moreover, future efforts in the development of single-cell sequencing technology will also emerge, which could focus on single-cell multimodal omics, such as genomic DNA–messenger RNA sequencing [44,45], genome and transcriptome sequencing [46], and single-cell methylome and transcriptome sequencing [47]. Currently, the areas of particular relevance to immuno-oncology based upon single-cell sequencing technology focus on the ability to track individual specific cell clones through paired sequencing of their cell receptor genes and high-dimensional single-cell spatial analysis [48]. High-dimensional single-cell sequencing technologies are likely to generate clinically relevant biomarker signatures in immuno-oncology and may be able to guide clinical decision making.

### Conclusions

The number of publications related to single-cell sequencing technology has increased dramatically since 2014. The United States led absolute productivity ranks by contributing approximately 60% of the total publications, followed by China, the United Kingdom, and Germany. Similarly, collaboration networks consisted mainly of institutes and authors from the above-mentioned countries. Moreover, in terms of the top 10 productive journals, the most cited journals, and journals with the highest average citation, there were 20 journals calculated in total. And most of them were considered as top-journals (impact factor＞10, Q1).Single-cell sequencing technology has made a large impact in various fields of biology, with a noticeable increase in publications annually in the last 5 years. We believe that the field of immunology might be a future research hotspot.

## Appendix

Multimedia Appendix 1 Distribution of top 10 productive authors.

Multimedia Appendix 2 Distribution by top journals and its citations.

Multimedia Appendix 3 Top 36 references with strongest citation bursts.

Multimedia Appendix 4 Top 15 keywords with the strongest citation bursts.

Multimedia Appendix 5 Clusters of immunotherapy in 2019.

## Abbreviations

WoSCC: Web of Science Core Collection

## References

[1] Bianconi E, Piovesan A, Facchin F, Beraudi A, Casadei R, Frabetti F, Vitale L, Pelleri MC, Tassani S, Piva F, Perez-Amodio S, Strippoli P, Canaider S (2013). An estimation of the number of cells in the human body. Ann Hum Biol.

[2] Wang Y, Navin NE (2015). Advances and applications of single-cell sequencing technologies. Mol Cell.

[3] Tang X, Huang Y, Lei J, Luo H, Zhu X (2019). The single-cell sequencing: new developments and medical applications. Cell Biosci.

[4] Wen L, Tang F (2018). Boosting the power of single-cell analysis. Nat Biotechnol.

[5] Stegle O, Teichmann SA, Marioni JC (2015). Computational and analytical challenges in single-cell transcriptomics. Nat Rev Genet.

[6] Tang F, Barbacioru C, Wang Y, Nordman E, Lee C, Xu N, Wang X, Bodeau J, Tuch BB, Siddiqui A, Lao K, Surani MA (2009). mRNA-Seq whole-transcriptome analysis of a single cell. Nat Methods.

[7] Dal Molin A, Di Camillo B (2019). How to design a single-cell RNA-sequencing experiment: pitfalls, challenges and perspectives. Brief Bioinform.

[8] Li J, Lin H, Hou R, Shen J, Li X, Xing J, He F, Wu X, Zhao X, Sun L, Fan X, Niu X, Liu Y, Liu R, An P, Qu T, Chang W, Wang Q, Zhou L, Li J, Wang Z, Jiao J, Wang Y, Wang G, Liang N, Liang J, Liang Y, Hou H, Shi Y, Yang X, Li J, Dang E, Yin G, Yang X, Zhang G, Gao Q, Fang X, Li X, Zhang K (2020). Multi-omics study in monozygotic twins confirm the contribution of de novo mutation to psoriasis. J Autoimmun.

[9] Zhang Q, He Y, Luo N, Patel SJ, Han Y, Gao R, Modak M, Carotta S, Haslinger C, Kind D, Peet GW, Zhong G, Lu S, Zhu W, Mao Y, Xiao M, Bergmann M, Hu X, Kerkar SP, Vogt AB, Pflanz S, Liu K, Peng J, Ren X, Zhang Z (2019). Landscape and dynamics of single immune cells in hepatocellular carcinoma. Cell.

[10] Wang Y, Niu Q, Zhang X, Liu L, Wang Y, Chen Y, Negi M, Figeys D, Li Y, Zhang T (2019). Exploring the effects of operational mode and microbial interactions on bacterial community assembly in a one-stage partial-nitritation anammox reactor using integrated multiomics. Microbiome.

[11] Lake BB, Chen S, Sos BC, Fan J, Kaeser GE, Yung YC, Duong TE, Gao D, Chun J, Kharchenko PV, Zhang K (2018). Integrative single-cell analysis of transcriptional and epigenetic states in the human adult brain. Nat Biotechnol.

[12] Rosenberg AB, Roco CM, Muscat RA, Kuchina A, Sample P, Yao Z, Graybuck LT, Peeler DJ, Mukherjee S, Chen W, Pun SH, Sellers DL, Tasic B, Seelig G (2018). Single-cell profiling of the developing mouse brain and spinal cord with split-pool barcoding. Science.

[13] Guo F, Li L, Li J, Wu X, Hu B, Zhu P, Wen L, Tang F (2017). Single-cell multi-omics sequencing of mouse early embryos and embryonic stem cells. Cell Res.

[14] Casasent AK, Schalck A, Gao R, Sei E, Long A, Pangburn W, Casasent T, Meric-Bernstam F, Edgerton ME, Navin NE (2018). Multiclonal invasion in breast tumors identified by topographic single cell sequencing. Cell.

[15] Suvà Mario L, Tirosh I (2019). Single-cell RNA sequencing in cancer: lessons learned and emerging challenges. Mol Cell.

[16] Zhang X, Liu L (2019). Applications of single cell RNA sequencing to research of stem cells. World J Stem Cells.

[17] Ren X, Kang B, Zhang Z (2018). Understanding tumor ecosystems by single-cell sequencing: promises and limitations. Genome Biol.

[18] Kiselev VY, Andrews TS, Hemberg M (2019). Challenges in unsupervised clustering of single-cell RNA-seq data. Nat Rev Genet.

[19] Agarwal A, Durairajanayagam D, Tatagari S, Esteves SC, Harlev A, Henkel R, Roychoudhury S, Homa S, Puchalt NG, Ramasamy R, Majzoub A, Ly KD, Tvrda E, Assidi M, Kesari K, Sharma R, Banihani S, Ko E, Abu-Elmagd M, Gosalvez J, Bashiri A (2016). Bibliometrics: tracking research impact by selecting the appropriate metrics. Asian J Androl.

[20] Keathley-Herring H, Van Aken E, Gonzalez-Aleu F, Deschamps F, Letens G, Orlandini PC (2016). Assessing the maturity of a research area: bibliometric review and proposed framework. Scientometrics.

[21] Chen C (2006). CiteSpace II: detecting and visualizing emerging trends and transient patterns in scientific literature. J Am Soc Inf Sci.

[22] van Eck NJ, Waltman L (2017). Citation-based clustering of publications using CitNetExplorer and VOSviewer. Scientometrics.

[23] Devos P, Ménard Joël (2020). Trends in worldwide research in hypertension over the period 1999-2018: a bibliometric study. Hypertension.

[24] van Eck Nees Jan, Waltman Ludo (2010). Software survey: VOSviewer, a computer program for bibliometric mapping. Scientometrics.

[25] van Eck NJ, Waltman L (2011). Text mining and visualization using VOSviewer. Arxiv.

[26] Ruiz Sebastián C, O’Ryan C (2001). Single-cell sequencing of dinoflagellate (Dinophyceae) nuclear ribosomal genes. Mol Ecol Notes.

[27] Bankevich A, Nurk S, Antipov D, Gurevich AA, Dvorkin M, Kulikov AS, Lesin VM, Nikolenko SI, Pham S, Prjibelski AD, Pyshkin AV, Sirotkin AV, Vyahhi N, Tesler G, Alekseyev MA, Pevzner PA (2012). SPAdes: a new genome assembly algorithm and its applications to single-cell sequencing. J Comput Biol.

[28] Navin N, Kendall J, Troge J, Andrews P, Rodgers L, McIndoo J, Cook K, Stepansky A, Levy D, Esposito D, Muthuswamy L, Krasnitz A, McCombie WR, Hicks J, Wigler M (2011). Tumour evolution inferred by single-cell sequencing. Nature.

[29] Patel AP, Tirosh I, Trombetta JJ, Shalek AK, Gillespie SM, Wakimoto H, Cahill DP, Nahed BV, Curry WT, Martuza RL, Louis DN, Rozenblatt-Rosen O, Suvà Mario L, Regev A, Bernstein BE (2014). Single-cell RNA-seq highlights intratumoral heterogeneity in primary glioblastoma. Science.

[30] Tirosh I, Izar B, Prakadan SM, Wadsworth MH, Treacy D, Trombetta JJ, Rotem A, Rodman C, Lian C, Murphy G, Fallahi-Sichani M, Dutton-Regester K, Lin J, Cohen O, Shah P, Lu D, Genshaft AS, Hughes TK, Ziegler CGK, Kazer SW, Gaillard A, Kolb KE, Villani A, Johannessen CM, Andreev AY, Van Allen EM, Bertagnolli M, Sorger PK, Sullivan RJ, Flaherty KT, Frederick DT, Jané-Valbuena Judit, Yoon CH, Rozenblatt-Rosen O, Shalek AK, Regev A, Garraway LA (2016). Dissecting the multicellular ecosystem of metastatic melanoma by single-cell RNA-seq. Science.

[31] Zeisel A, Muñoz-Manchado Ana B, Codeluppi S, Lönnerberg Peter, La Manno G, Juréus Anna, Marques S, Munguba H, He L, Betsholtz C, Rolny C, Castelo-Branco G, Hjerling-Leffler J, Linnarsson S (2015). Brain structure. cell types in the mouse cortex and hippocampus revealed by single-cell RNA-seq. Science.

[32] Kolodziejczyk AA, Kim JK, Svensson V, Marioni JC, Teichmann SA (2015). The technology and biology of single-cell RNA sequencing. Mol Cell.

[33] Li A, Tang X (2017). Foundation support for studies on single-cell sequencingits application in USA, Britain and China. Chin J Med Lib Info Sci.

[34] Metzker ML (2005). Emerging technologies in DNA sequencing. Genome Res.

[35] Xu X, Hou Y, Yin X, Bao L, Tang A, Song L, Li F, Tsang S, Wu K, Wu H, He W, Zeng L, Xing M, Wu R, Jiang H, Liu X, Cao D, Guo G, Hu X, Gui Y, Li Z, Xie W, Sun X, Shi M, Cai Z, Wang B, Zhong M, Li J, Lu Z, Gu N, Zhang X, Goodman L, Bolund L, Wang J, Yang H, Kristiansen K, Dean M, Li Y, Wang J (2012). Single-cell exome sequencing reveals single-nucleotide mutation characteristics of a kidney tumor. Cell.

[36] Wang J, Fan HC, Behr B, Quake SR (2012). Genome-wide single-cell analysis of recombination activity and de novo mutation rates in human sperm. Cell.

[37] Evrony GD, Cai X, Lee E, Hills LB, Elhosary PC, Lehmann HS, Parker JJ, Atabay KD, Gilmore EC, Poduri A, Park PJ, Walsh CA (2012). Single-neuron sequencing analysis of L1 retrotransposition and somatic mutation in the human brain. Cell.

[38] Ramsköld Daniel, Luo S, Wang Y, Li R, Deng Q, Faridani OR, Daniels GA, Khrebtukova I, Loring JF, Laurent LC, Schroth GP, Sandberg R (2012). Full-length mRNA-seq from single-cell levels of RNA and individual circulating tumor cells. Nat Biotechnol.

[39] Rinke C, Schwientek P, Sczyrba A, Ivanova NN, Anderson IJ, Cheng J, Darling A, Malfatti S, Swan BK, Gies EA, Dodsworth JA, Hedlund BP, Tsiamis G, Sievert SM, Liu W, Eisen JA, Hallam SJ, Kyrpides NC, Stepanauskas R, Rubin EM, Hugenholtz P, Woyke T (2013). Insights into the phylogeny and coding potential of microbial dark matter. Nature.

[40] Shalek AK, Satija R, Adiconis X, Gertner RS, Gaublomme JT, Raychowdhury R, Schwartz S, Yosef N, Malboeuf C, Lu D, Trombetta JJ, Gennert D, Gnirke A, Goren A, Hacohen N, Levin JZ, Park H, Regev A (2013). Single-cell transcriptomics reveals bimodality in expression and splicing in immune cells. Nature.

[41] Nagano T, Lubling Y, Stevens TJ, Schoenfelder S, Yaffe E, Dean W, Laue ED, Tanay A, Fraser P (2013). Single-cell Hi-C reveals cell-to-cell variability in chromosome structure. Nature.

[42] Peng C, He M, Cutrona SL, Kiefe CI, Liu F, Wang Z (2020). Theme trends and knowledge structure on mobile health apps: bibliometric analysis. JMIR Mhealth Uhealth.

[43] Zheng Y, Chen Z, Han Y, Han L, Zou X, Zhou B, Hu R, Hao J, Bai S, Xiao H, Li WV, Bueker A, Ma Y, Xie G, Yang J, Chen S, Li H, Cao J, Shen L (2020). Immune suppressive landscape in the human esophageal squamous cell carcinoma microenvironment. Nat Commun.

[44] Zhu C, Preissl S, Ren B (2020). Single-cell multimodal omics: the power of many. Nat Methods.

[45] Dey SS, Kester L, Spanjaard B, Bienko M, van Oudenaarden A (2015). Integrated genome and transcriptome sequencing of the same cell. Nat Biotechnol.

[46] Macaulay IC, Haerty W, Kumar P, Li YI, Hu TX, Teng MJ, Goolam M, Saurat N, Coupland P, Shirley LM, Smith M, Van der Aa N, Banerjee R, Ellis PD, Quail MA, Swerdlow HP, Zernicka-Goetz M, Livesey FJ, Ponting CP, Voet T (2015). Nat Methods.

[47] Angermueller C, Clark SJ, Lee HJ, Macaulay IC, Teng MJ, Hu TX, Krueger F, Smallwood S, Ponting CP, Voet T, Kelsey G, Stegle O, Reik W (2016). Parallel single-cell sequencing links transcriptional and epigenetic heterogeneity. Nat Methods.

[48] Gohil SH, Iorgulescu JB, Braun DA, Keskin DB, Livak KJ (2021). Applying high-dimensional single-cell technologies to the analysis of cancer immunotherapy. Nat Rev Clin Oncol.

