# Supplementary material for: Characterization of Global Research Trends and Prospects on Single-Cell Sequencing Technology: Bibliometric Analysis
Source: J Med Internet Res. 2021 Aug 10;23(8):e25789. doi: 10.2196/25789 (PMC8386406; doi:10.2196/25789)
Supplement: Multimedia Appendix 1 [file jmir_v23i8e25789_app1.docx]

**Supplementary file 1. Distribution of top 10 productive authors**

| **Productive authors** | **Institutes** | **Country** | **Documents** | **Percentage (%)** | **Citations** | **Total link strength** |
| --- | --- | --- | --- | --- | --- | --- |
| Teichmann, SA. | University of Cambridge; Wellcome Trust Sanger Institute, Wellcome Genome Campus, Hinxton, UK | UK | 40 | 1.6% | 2625 | 40 |
| Regev, A. | Massachusetts Institute of Technology | USA | 38 | 1.5% | 4812 | 38 |
| Marioni, JC. | Cancer Research UK Cambridge Institute, University of Cambridge | UK | 38 | 1.5% | 2852 | 38 |
| Tang, FC. | Peking University | China | 26 | 1.0% | 1717 | 26 |
| Shalek, AK. | Institute for Medical Engineering and Science (IMES), MIT, Cambridge, MA, USA. | USA | 25 | 1.0% | 2829 | 25 |
| Sandberg, R. | Department of Cell and Molecular Biology, Karolinska Institute, Stockholm, Sweden | Sweden | 25 | 1.0% | 2033 | 25 |
| Linnarsson, S. | Department of Medical Biochemistry and Biophysics, Karolinska Institutet, Stockholm, Sweden. | Sweden. | 24 | 1.0% | 3693 | 24 |
| Amit, I. | Department of Immunology, Weizmann Institute of Science, Rehovot, Israel. | Israel | 21 | 0.8% | 1383 | 21 |
| Satija, R. | New York University, Center for Genomics and Systems Biology, New York, USA | USA | 19 | 0.8% | 3276 | 19 |
| Quake, SR. | Department of Bioengineering, Stanford University | USA | 19 | 0.8% | 1634 | 19 |
