# Supplementary material for: Characterization of Global Research Trends and Prospects on Single-Cell Sequencing Technology: Bibliometric Analysis
Source: J Med Internet Res. 2021 Aug 10;23(8):e25789. doi: 10.2196/25789 (PMC8386406; doi:10.2196/25789)
Supplement: Multimedia Appendix 2 [file jmir_v23i8e25789_app2.docx]

| **Rank** | **Top productive journals** | | |  | **Top-cited journals** | | |  | **Top average citations** | | |
| --- | --- | --- | --- | --- | --- | --- | --- | --- | --- | --- | --- |
|  | **Journals (IF&Q)** | **Total number**  **(n)** | **Percentage (%)** |  | **Journals**  **(IF&Q)** | **Total number**  **(n)** | **Total Citations** |  | **Journals**  **(IF&Q)** | **Total number**  **(n)** | **Average times** |
| 1 | Nature Communications  (11.878, Q1) | 127 | 5.1% |  | Nature Methods  (28.467, Q1) | 55 | 1764 |  | Nature Biotechnology  (31.864, Q1) | 28 | 56.5 |
| 2 | Genome Biology  (14.028, Q1) | 84 | 3.4% |  | Science  (41.063, Q1) | 41 | 1691 |  | Science  (41.063, Q1) | 41 | 41.2 |
| 3 | Cancer Research  (8.387, Q1) | 70 | 2.8% |  | Nature Biotechnology  (31.864, Q1) | 28 | 1581 |  | Nature Structural & Molecular Biology  (12.109, Q1) | 3 | 38.7 |
| 4 | Cell Reports  (7.815, Q1) | 66 | 2.7% |  | Nature  (43.070, Q1) | 53 | 1380 |  | Nature Reviews Immunology  (44.019, Q1) | 1 | 38.0 |
| 5 | Bioinformatics  (4.531, Q1) | 66 | 2.7% |  | Genome Biology  (14.028, Q1) | 84 | 1316 |  | Nature Methods  (28.467, Q1) | 55 | 32.1 |
| 6 | Cell  (36.216, Q1) | 60 | 2.4% |  | Cell  (36.216, Q1) | 60 | 862 |  | Nature Reviews Genetics  (43.704, Q1) | 9 | 31.0 |
| 7 | Scientific Reports  (4.011, Q1) | 57 | 2.3% |  | Nature Communications  (11.878, Q1) | 127 | 488 |  | Nature Protocols  (11.334, Q1) | 10 | 31.0 |
| 8 | Nature Methods  (28.467, Q1) | 55 | 2.2% |  | Genome Research  (9.944, Q1) | 32 | 485 |  | Methods  (3.782, Q2) | 2 | 27.0 |
| 9 | Nature  (43.070, Q1) | 53 | 2.1% |  | Cell Reports  (7.815, Q1) | 66 | 465 |  | Nature  (43.070, Q1) | 53 | 26.0 |
| 10 | P NATL ACAD SCI USA (9.580, Q1) | 47 | 1.9% |  | Bioinformatics  (4.531, Q1) | 66 | 349 |  | Molecular Cell  (14.548, Q1) | 14 | 20.4 |
| P NATL ACAD SCI USA: Proceedings of the National Academy of Science of the United States of America;  IF&Q: Journal Impact Factor (JIF) and JIF Quartile, which was based on the data of 2019.  BMC GENOMICS 3.501,Q2 | | | | | | | | | | | |

**Supplementary file 2. Distribution by top journals and its citations**
