# Supplementary figures and images for: Characterization of Global Research Trends and Prospects on Single-Cell Sequencing Technology: Bibliometric Analysis
Source: J Med Internet Res. 2021 Aug 10;23(8):e25789. doi: 10.2196/25789 (PMC8386406; doi:10.2196/25789)

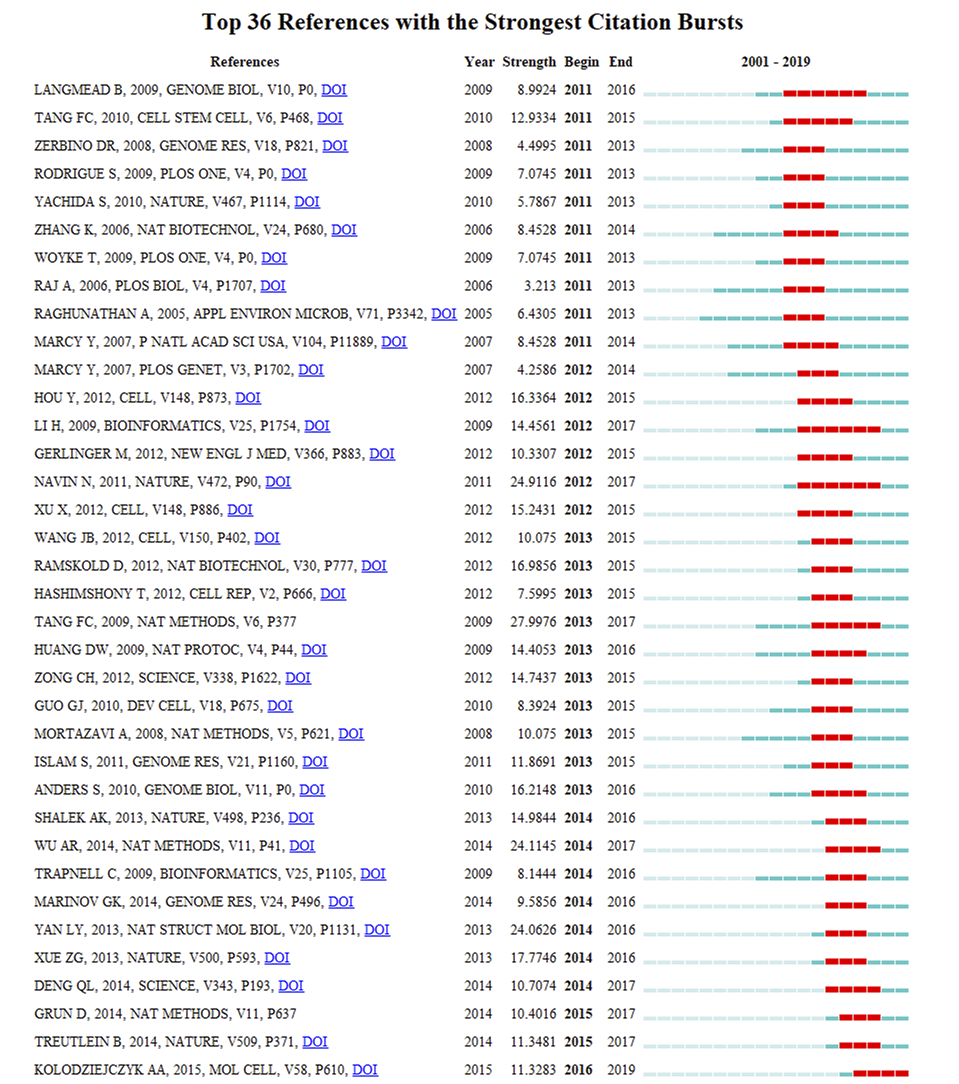

Supplement: Multimedia Appendix 3 [file jmir_v23i8e25789_app3.png]

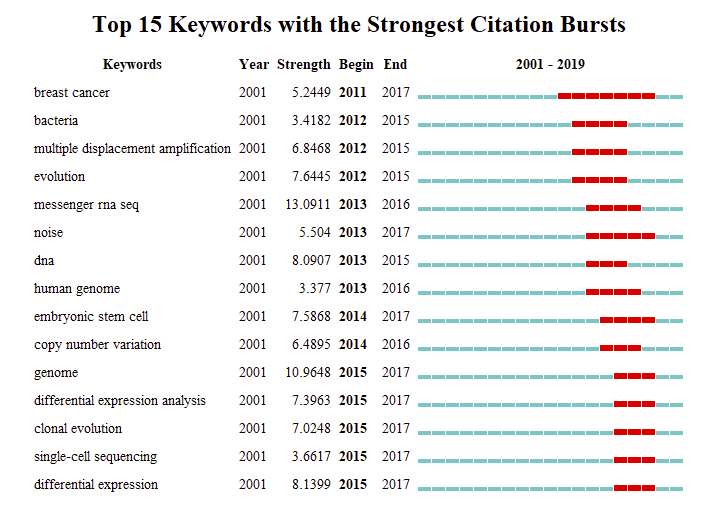

Supplement: Multimedia Appendix 4 [file jmir_v23i8e25789_app4.png]

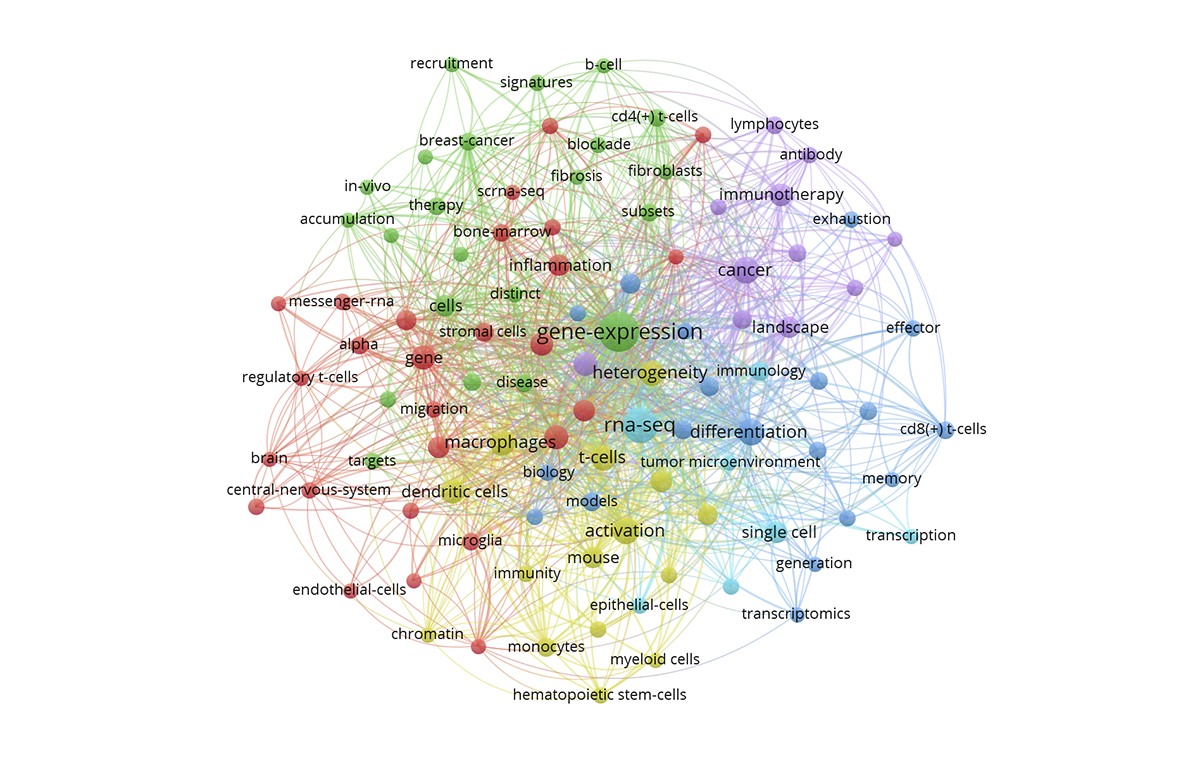

Supplement: Multimedia Appendix 5 [file jmir_v23i8e25789_app5.png]
